# Supplementary figures and images for: Active Learning for Rapid Targeted Synthesis of Compositionally Complex Alloys
Source: Materials (Basel). 2024 Aug 14;17(16):4038. doi: 10.3390/ma17164038 (PMC11355945; doi:10.3390/ma17164038)

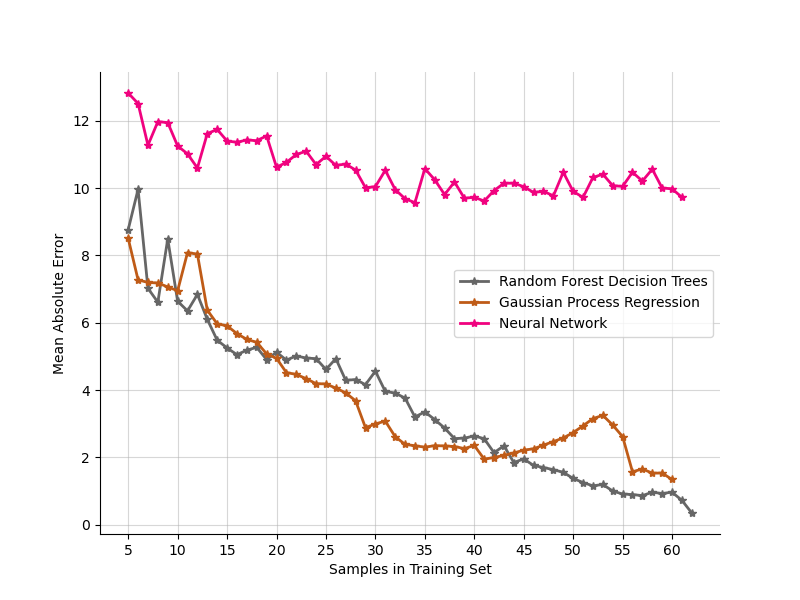

Supplement: Supplementary file 1 [file materials-17-04038-s001.zip › figures/Supplementary Figure S1.png]

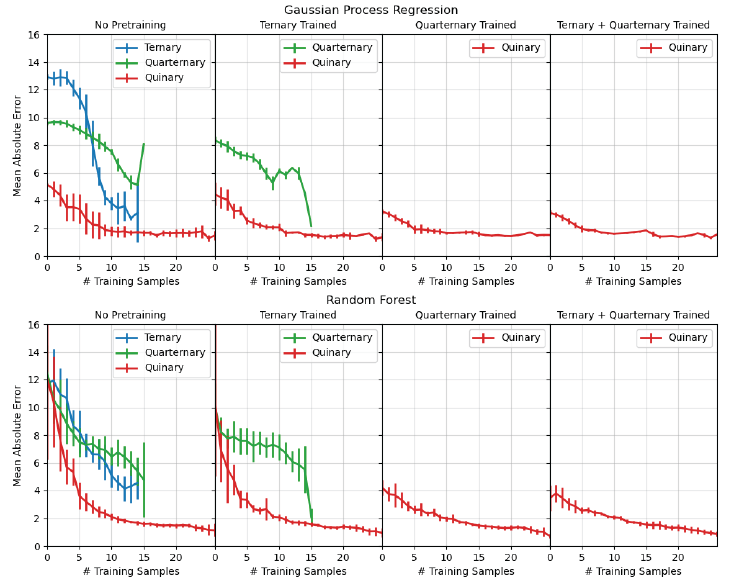

Supplement: Supplementary file 1 [file materials-17-04038-s001.zip › figures/Supplementary Figure S2.png]

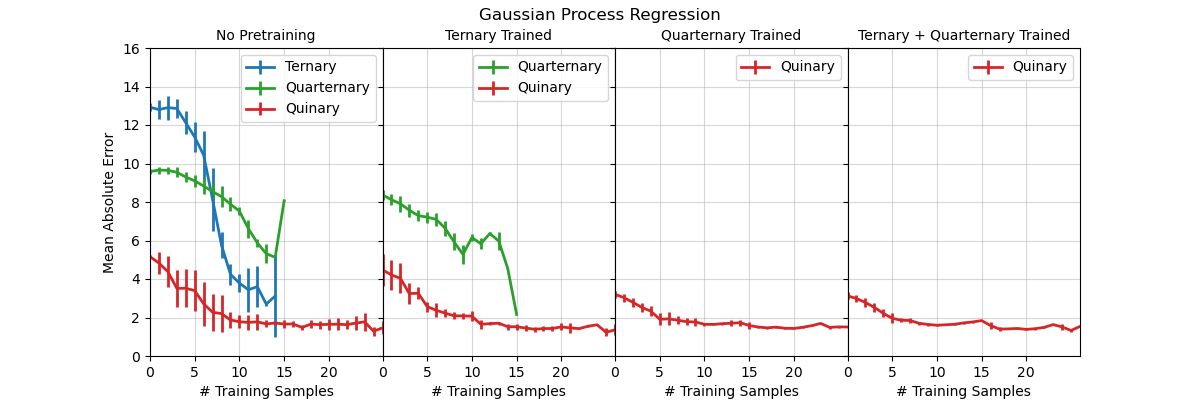

Supplement: Supplementary file 1 [file materials-17-04038-s001.zip › figures/Supplementary Figure S3.png]

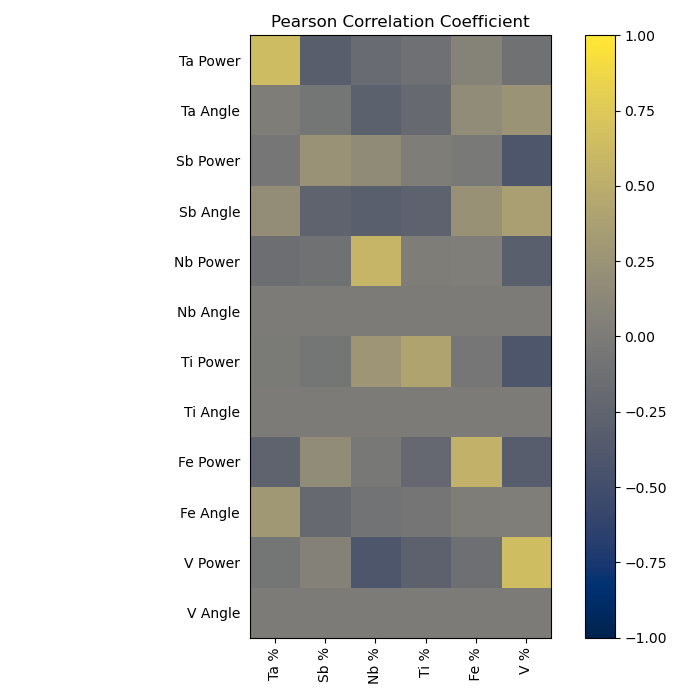

Supplement: Supplementary file 1 [file materials-17-04038-s001.zip › figures/Supplementary Figure S4.png]

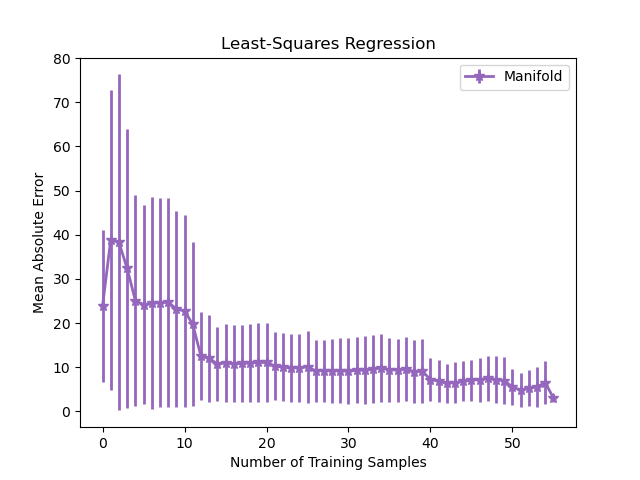

Supplement: Supplementary file 1 [file materials-17-04038-s001.zip › figures/Supplementary Figure S5.png]

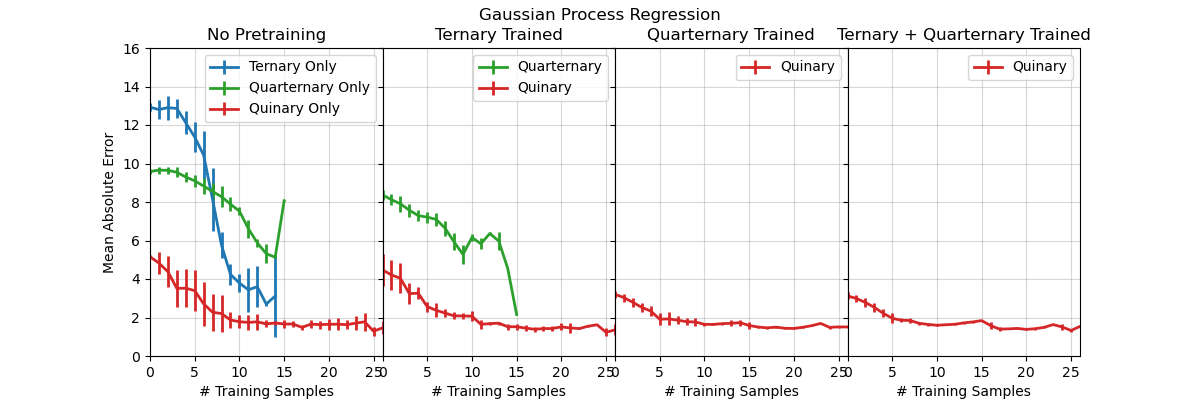

Supplement: Supplementary file 1 [file materials-17-04038-s001.zip › figures/Supplementary Figure S6.png]
